# Cuticular hydrocarbon profiles differ between ant body parts: implications for communication and our understanding of CHC diffusion

**DOI:** 10.1093/cz/zoab012

**Published:** 2021-02-11

**Authors:** Philipp P Sprenger, Lisa J Gerbes, Jacqueline Sahm, Florian Menzel

**Affiliations:** 1 Institute of Organismic and Molecular Evolution (iomE), Johannes Gutenberg-University Mainz, Hanns-Dieter-Hüsch-Weg 15, Mainz, 55128, Germany; 2 Department of Evolutionary Animal Ecology, University of Bayreuth, Universitätsstraße 30, Bayreuth, 95477, Germany

**Keywords:** nestmate recognition, Formicidae, eusocial insects, melting temperature, cuticle regionalization, pheromones

## Abstract

Insect cuticular hydrocarbons (CHCs) serve as communication signals and protect against desiccation. They form complex blends of up to 150 different compounds. Due to differences in molecular packing, CHC classes differ in melting point. Communication is especially important in social insects like ants, which use CHCs to communicate within the colony and to recognize nestmates. Nestmate recognition models often assume a homogenous colony odor, where CHCs are collected, mixed, and redistributed in the postpharyngeal gland (PPG). Via diffusion, recognition cues should evenly spread over the body surface. Hence, CHC composition should be similar across body parts and in the PPG. To test this, we compared CHC composition among whole-body extracts, PPG, legs, thorax, and gaster, across 17 ant species from 3 genera. Quantitative CHC composition differed between body parts, with consistent patterns across species and CHC classes. Early-melting CHC classes were most abundant in the PPG. In contrast, whole body, gaster, thorax, and legs had increasing proportions of CHC classes with higher melting points. Intraindividual CHC variation was highest for rather solid, late-melting CHC classes, suggesting that CHCs differ in their diffusion rates across the body surface. Our results show that body parts strongly differ in CHC composition, either being rich in rather solid, late-melting, or rather liquid, early-melting CHCs. This implies that recognition cues are not homogenously present across the insect body. However, the unequal diffusion of different CHCs represents a biophysical mechanism that enables caste differences despite continuous CHC exchange among colony members.

Cuticular hydrocarbons (CHCs) cover the body surface of nearly all insects (Blomquist and Bagnères 2010), which is the most diverse and abundant animal group on earth. The CHC layer protects against desiccation and carries communication signals. In many solitary insects, CHCs serve as contact sex pheromone and can be under sexual selection (Steiger et al. 2013; Steiger and Stökl 2014). Especially in ants and other social insects, communication via CHCs is vital for the organization of social life, which is reflected in their highly diverse CHC profiles (Martin and Drijfhout 2009a; Helmkampf et al. 2015; Sprenger and Menzel 2020). In ants, CHCs are vital not only to communicate task, fertility, and reproductive status within the colony (Greene and Gordon 2003; Holman et al. 2010; Liebig 2010; Leonhardt et al. 2016), but also to distinguish nestmates from non-nestmates (van Zweden and d’Ettorre 2010).

Nestmate recognition is thought to be based on a homogenous colony odor that may or may not encompass all hydrocarbons. According to the *Gestalt* model, this uniform odor (*Gestalt* odor) is achieved by continuous CHC exchange among workers. This exchange happens via allogrooming, that is, licking and trophallaxis, that is, exchange of fluids (LeBoeuf et al. 2016). Here, the postpharyngeal gland (PPG) plays a vital role in ants. Being located close to the mouthparts, individual ants use it to store hydrocarbons not only from themselves (obtained via self-grooming), but also those from CHC exchange among nestmates (Soroker et al. 1994; Boulay et al. 2000; LeBoeuf et al. 2016). The resulting mixture is then redistributed on the body surface during self- and allogrooming (Soroker et al. 1995; Lahav et al. 1999; Soroker and Hefetz 2000), which is supposed to homogenize interindividual differences and create a uniform colony odor. When 2 social insects meet, they perceive each other’s profile via their antennae and compare it to neuronal templates to distinguish nestmates from non-nestmates (van Zweden and d’Ettorre 2010). Note that the PPG does not produce CHCs, but serves as an intermittent organ for CHC storage and mixing in ants. CHCs are produced in oenocytes, which are associated either to epidermal cells or to the fat body. From there, they are transported to the cuticle via lipophorins (Schal et al. 1998b; Fan et al. 2004; Bagnères and Blomquist 2010), where they can be perceived or taken up by other individuals. In other social insects, CHCs are homogenized in different ways: in bees and wasps, CHC homogenization is probably achieved via contact to nest paper or comb wax (van Zweden and d’Ettorre 2010), whereas mechanisms of CHC exchange are largely unknown in termites (Bagnères and Hanus 2015). According to the *Gestalt* model, all recognition cues applied via allogrooming or from other sources should evenly spread over the body surface via diffusion and, thus, be similar across the body surface and in the PPG. This tacitly assumes that all hydrocarbons are uniformly spread over an individual’s body surface, and also requires that all hydrocarbons can be taken up via grooming equally well.

However, CHC profiles are complex blends of up to 150 hydrocarbons that differ in melting point and phase behavior, and form a heterogenous mixture of solid and liquid phases on the body surface (Gibbs 2002; Menzel et al. 2019). The main hydrocarbon classes in insects include *n-*alkanes, monomethyl alkanes, dimethyl alkanes, alkenes, and alkadienes (Sprenger and Menzel 2020; Figure 1A). Generally, melting temperature (T_m_) is highest in *n*-alkanes, which pack most tightly due to strong van der Waals forces (Figure 1B). The presence of methyl branches and double bonds in the molecule drastically lowers T_m_ because molecular packing is less tight. This effect is much stronger than the increase of T_m_ with chain length (Gibbs 1995; Gibbs and Pomonis 1995; Gibbs and Rajpurohit 2010). The reduction in melting temperature also depends on number and position of methyl branches and unsaturations (Gibbs and Pomonis 1995; Gibbs 1998): Methyl-branched hydrocarbons with rather terminal methyl groups (e.g., 3- or 5-methyl alkanes) can still aggregate with large parts of their carbon backbones and thus usually have a higher T_m_ than internally-branched hydrocarbons (such as 13-, 15-methyl alkanes), which consequently melt earlier than terminally branched ones (Gibbs and Pomonis 1995). The different melting ranges of CHC classes affect waterproofing ability, as earlier studies related cuticular water loss to CHC melting (Rourke and Gibbs 1999). Moreover, they translate to differences in viscosity and diffusion rate across the insect body (Sprenger et al. 2018; Menzel et al. 2019), which may lead to different CHC composition among body parts. Indeed, recent studies provided evidence for such body part-specific CHC differences: melting temperatures differed between body parts in *Drosophila melanogaster* adults and other insects (Wang et al. 2016b, 2017; but also see Gibbs and Crowe 1991), which suggests differences in CHC composition. In social insects, CHC differences between body parts were found in the meat ant *Iridomyrmex purpureus* (Wang et al. 2016a, 2019). To our knowledge, these 2 studies on *I. purpureus* are the only ones testing differences of CHC profiles between body parts in ants.

**Figure 1.**
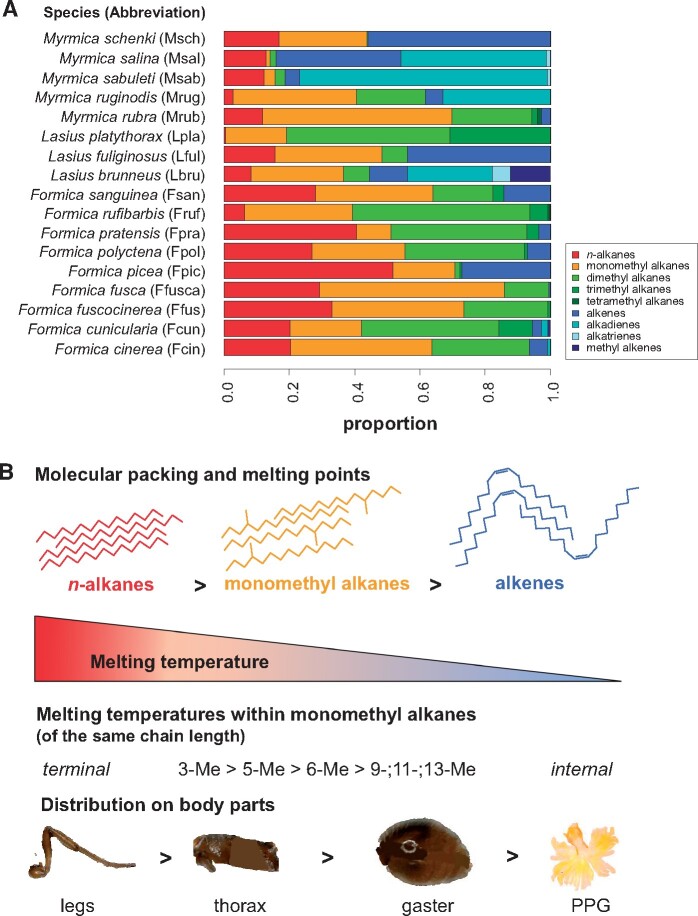
CHC composition of the ant species in this study (**A**) and physical properties of CHCs (**B**). (A) Each bar plot shows the average substance class composition of the 17 species studied here. The data are based on whole-body extracts. While quantitative composition can vary among conspecifics, the qualitative composition is highly species-specific (Sprenger and Menzel 2020). (B) Overview of molecular packing and the resulting melting temperatures for different CHC classes, as well as the melting temperatures within monomethyl alkanes of the same chain length and the CHC distribution on body parts. Photos of *M. rubra* (legs, thorax, gaster) by P. Sprenger, photo of the PPG by L. Gerbes.

Here, we investigated whether CHC composition differs among body parts (including the PPG), which might influence the biological functions of the CHC layer. Since the diffusion of CHCs may depend on their composition (i.e., on their phase behavior), we additionally compared the degree of CHC variation across ant species with varying composition. In particular, we asked 2 questions: 1) How do CHCs differ between body parts, and are these differences species-specific or consistent across species? 2) Do these differences depend on the biophysical properties, that is, the fluidity, of the CHC layer? We hypothesized that more viscous or solid CHCs (i.e., *n*-alkanes and terminally branched monomethyl alkanes), which have better waterproofing capacities (Figure 1B), should be present in higher proportions on body parts like legs which have high surface-to-volume ratios and thus are more prone to desiccation. Furthermore, more liquid CHCs important for the chemical communication (e.g., dimethyl alkanes, alkenes, and alkadienes; Gibbs 2002) can be shared easier between nestmates and thus should be present in higher proportions in the PPG. We first analyzed body part differentiation in one ant species, *Myrmica rubra*, and then compared it across 16 further ant species.

## Materials and Methods

### Study species

We examined the CHC profiles of 17 Central European ant species of the genera *Formica*, *Myrmica*, and *Lasius*. Specimen was collected at the Botanical Gardens of the University of Bayreuth (Germany), in Mainz (Germany), Darmstadt (Germany), or Eupen (Belgium) (Supplementary Table S1) and identified according to Seifert (2007). Workers of all species were freeze-killed at −20°C and kept at this temperature until further processing.

### Dissection of body parts and CHC extraction

First, we analyzed intraindividual CHC differences in 22 individuals of *M. rubra* which originated from 10 independent colonies. CHCs were extracted from 4 different body parts of the same individuals: the PPG, gaster (G), thorax (T), and legs (L) (*N = *22 × 4 body parts). The PPGs were removed from the ants’ heads through the mouth using micro tweezers and afterward put in glass vials filled with *n*-hexane. After dissection of the PPG, the gasters, thoraces, and legs were separated and extracted in *n*-hexane for 10 min. Subsequently, we concentrated the extracts under a gentle stream of nitrogen. We cannot exclude that samples might have been contaminated with extruding hemolymph or gland extract. However, this bias should be minor because, in most cases, we did not observe any extruding hemolymph (except for PPGs). Moreover, we dissected under water; hemolymph is an aqueous solution and hence quickly dilute in the water. We took care to remove any water before placing body parts or PPGs into hexane.

Second, for an interspecific comparison of 16 ant species, the CHCs were either extracted from the whole body (*N = *42) or from the 4 different body parts (*N = *54 individuals × 4 body parts) as described above (Supplementary Table S1). We had to omit 12 samples due to very low CHC concentration, resulting in a total of *N = *246 samples (Supplementary Table S1).

### GC-MS analysis

The CHC extracts were analyzed with gas chromatography-mass spectrometry (GC-MS). The GC (7890A, Agilent Technologies, Santa Clara, CA, USA) was equipped with a Zebron Inferno ZB5-HT column (Phenomenex Ltd., Aschaffenburg, Germany). Helium was used as carrier gas at a flow rate of 1.2 mL/min. We injected 2 µL of each sample in splitless mode at 60°C. Temperature was held constant for 2 min, then increased with 60°C/min to 200°C and afterward with 4°C/min until the maximum temperature of 320°C. This temperature was again held constant for another 10 min. Hydrocarbon molecules were then transferred to a mass selective detector (5975 C, Agilent Technologies) and fragmented with an ionization voltage of 70 eV. Fragments in the range of 40–550 m/z were detected and subsequently used for substance identification. To identify the CHCs, we used retention indices and diagnostic ions (Carlson et al. 1998).

For quantification, all chromatogram peaks were integrated manually in *MSD ChemStation* (E.02.02.1431, Agilent Technologies). Relative proportions were calculated in *Microsoft Excel*. Trace substances below 0.5% of the total profile and nonhydrocarbons were removed from the dataset. Afterward, the profiles were standardized to 100%.

### Statistical analyses dataset 1: Body part differentiation in *M. rubra*

To compare the CHC profiles between body parts in *M. rubra*, we first ordinated the samples using nonmetric multidimensional scaling (NMDS) based on Bray–Curtis dissimilarities (R command *metaMDS*, R package *vegan*; Oksanen et al. 2019). Furthermore, we calculated 95% confidence intervals around the centroids of each body part (R command *ordiellipse*, R package *ellipse*; Murdoch and Chow 2020). To compare the overall CHC profile between body parts, we used a permutational ANOVA (PERMANOVA; (software PRIMER 6 v6.1.14 and PERMANOVA+ v1.0.4; Primer-E Ltd.) with body part as fixed factor and colony and individual (nested in colony) as random factors.

Afterward, we analyzed the differences between body parts in different substance classes (*n*-alkanes, mono-, di-, and trimethyl alkanes and alkenes; alkadienes and tetramethyl alkanes do not occur in *M. rubra*) and homologous series of mono- and dimethyl alkanes (3-methyl, 5-methyl, and internally-branched methyl alkanes) using linear mixed effects models (R command *lme*, R package *nlme*; Pinheiro et al. 2016). Internally-branched alkanes (methyl group at position 8 or higher) are hardly separable using gas chromatography. For a given chain length, melting temperature decreases with methyl group position, but the differences are small for positions above 6 (Gibbs and Pomonis 1995), which is why we pooled these methyl alkanes. For the models, we used arcus-sinus square root-transformed proportions of the substance classes as response variable, body parts as fixed factor, and the individual ID nested in colony ID as random factor.

### Statistical analyses dataset 2: Interspecific comparison

For all interspecific comparisons, we summed CHC profiles up according to CHC class (*n*-alkanes, mono-, di-, tri-, and tetramethyl alkanes, alkenes, and alkadienes) and, for mono- and dimethyl alkanes, according to homologous series, that is, 3-methyl, 5-methyl (or 3, x-dimethyl and 5, x-dimethyl, respectively) and internally-branched methyl alkanes. Moreover, we calculated the average chain lengths for each body part. Due to the strong impact of CHC class on melting points, calculating overall chain lengths would be misleading—for example, branched alkanes are often longer (on average) than unbranched ones, but nevertheless melt earlier (Gibbs 2002). However, average chain lengths can be meaningful when focusing on a single substance class with not molecular heterogeneity. Therefore, we analyzed the average chain length of the *n*-alkanes in a sample.

To investigate overall differences between the whole-body CHC profiles and those of the different body parts in the 16 different species, we calculated the Bray–Curtis dissimilarities between the profiles (based on the above CHC classes) and visualized them using NMDS. Subsequently, we calculated the 95% confidence interval ellipses for each body part as described above. Similar to Dataset 1, we compared CHC composition using a PERMANOVA with species and body part as fixed effects and individual (nested in species) as random effect.

Subsequently, we tested differences between body parts were consistent across species. To this end, we first standardized all proportions of a CHC class (or the *n-*alkane chain length, respectively) on a given body part *x* by its average proportion in the whole-body extract using $\frac{p_{x}-p_{wb}}{p_{wb}}$, where *p_x_* is the proportion of a CHC class on body part *x* and *p_wb_* is the proportion of this CHC class in the average over all whole-body extracts of this species. Applying this formula resulted in the relative difference in a certain substance class between a body part and the whole body. For example, a value of 0.5 for *n-*alkanes in legs would mean that legs contained 50% more *n-*alkanes compared with the entire body, whereas a value of −0.8 would mean that they contained 80% less *n*-alkanes; a value of 0 would mean that the proportion of *n-*alkanes in legs and in whole-body extracts is the same. We used these relative differences as response variables in 2 types of linear mixed effects models (R command *lme*, R package *nlme*; Pinheiro et al. 2016): 1) We tested the deviation from 0 for the different body parts to investigate if they differ from the whole-body profiles and 2) we used the body parts as fixed factors to analyze differences between them. In both sets of regression models, we used individual ID nested in species as random factor.

In the second analysis, we investigated if the degree of intraindividual variability depended on the CHC composition of the respective species. The underlying reasoning was that CHC profiles with more fluid CHCs (like alkenes) would allow a higher CHC diffusion, resulting in lower variation between body parts. We only used individuals for which we had CHC information on all body parts (*N = *47). First, we calculated the coefficient of variation (CV) of CHC proportions across body parts per individual, separately for the proportions of *n*-alkanes, monomethyl alkanes, dimethyl alkanes, alkenes, and alkadienes. Subsequently, we used the sum of these separate CVs per individual as a single response variable for 6 different linear mixed effects models: We tested if the CV per individual was affected by the total proportion of either of the 5 substance classes mentioned above and (as species often either contain many dimethyl alkanes or many unsaturated CHCs) a combination of the most fluid substance classes (dimethyl alkanes + alkenes + alkadienes) as fixed effects. In each model, we used species ID as random factor.

All statistical analyses were conducted using *R* version 4.0.2 (R Core Team 2020) and the packages mentioned above.

## Results

### Dataset 1: Body part differentiation in *M. rubra*

**Figure 2.**
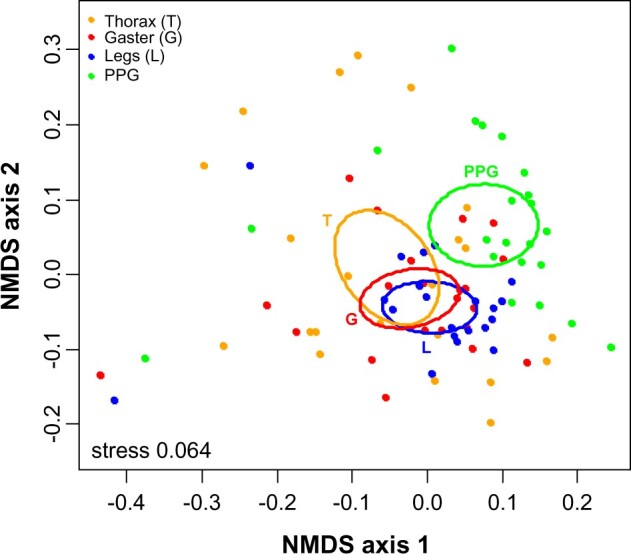
NMDS ordinations of the CHC profiles of different body parts in *M. rubra*. Each dot represents 1 CHC extract either of legs, thorax, gaster, or PPG. Ellipses represent the 95% confidence intervals around the centroids of the respective body parts.

CHC profiles strongly differed among 4 different body parts of 22 *M. rubra* individuals (PERMANOVA: pseudo-F_3_ = 4.6, *P = *0.0006). In the ordination, the PPG was different from all other body parts (all pairwise *t* > 2.4, *P < *0.01). However, the overall profiles of gasters, thoraces, and legs did not differ from each other (Figure 2; all pairwise *t* < 1.2, *P > *0.25).

**Figure 3.**
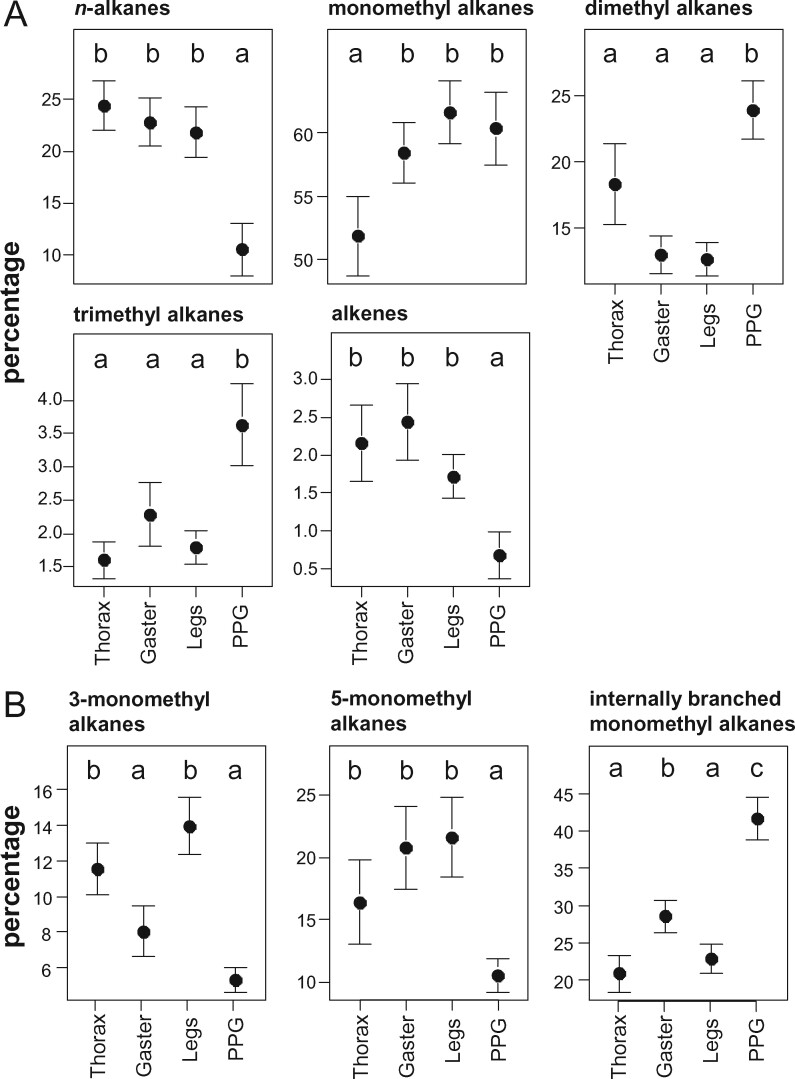
Body part-specific differences in CHC substance classes (**A**) and homologous series of methyl alkanes (**B**) in *M. rubra*. Each plot shows means ± SE of the proportion of 1 substance class (A) or homologous series (B) per body part. Different letters indicate statistically different values based on the results of linear mixed effects models.

In particular, the PPG contained lower proportions of *n*-alkanes (LME: χ^2^_3_ = 70.37, *P < *0.0001) and alkenes (χ^2^_3_ = 20.50, *P = *0.0001; Figure 3A), whereas the proportion of dimethyl alkanes (χ^2^_3_ = 22.77, *P < *0.0001; Figure 3A) and trimethyl alkanes (χ^2^_3_ = 19.40, *P = *0.0002; Figure 3A) was highest in the PPGs compared with other body parts. Thoraces contained slightly lower proportions of monomethyl alkanes compared with the other body parts (χ^2^_3_ = 10.97, *P = *0.012).

The proportions of various homologous series of mono- and dimethyl alkanes also differed between the body parts in *M. rubra*: In the PPG, the proportions of 3- and 5-methyl alkanes were lowest (χ^2^_3_ = 32.74, *P < *0.0001; χ^2^_3_ = 20.02, *P = *0.0002; Figure 3B), whereas those of internally-branched methyl alkanes were highest (χ^2^_3_ = 63.26, *P < *0.0001; Figure 3B). Surprisingly, the proportion of 3-methyl alkanes was lower on the gaster of *M. rubra* than on thorax and legs (Figure 3B). Although body parts did not differ in the proportions of 3, x-dimethyl alkanes (χ^2^_3_ = 6.15, *P = *0.10), those of 5, x- and internally-branched dimethyl alkanes were higher in the PPGs (χ^2^_3_ = 14.24, *P = *0.0026; χ^2^_3_ = 24.23, *P < *0.0001; Supplementary Figure S1A). The proportion of internally-branched dimethyl alkanes was also higher on the thoraces than on gasters and legs of *M. rubra* (Supplementary Figure S1A).

### Dataset 2: Interspecific comparison

**Figure 4.**
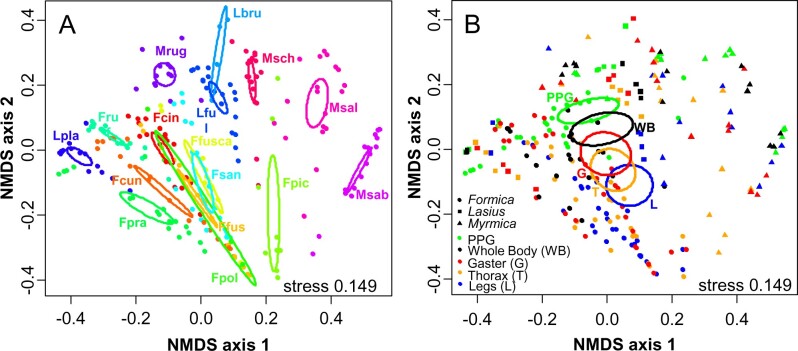
NMDS ordinations of the CHC profiles of 16 Central European ant species. Each dot represents 1 CHC extract either of legs, thorax, gaster, PPG, or whole body. Dots are colored after species (**A**) (see Figure 1A for abbreviations) or body part (**B**). In (B), different symbols reflect the 3 ant families examined in this comparison. Ellipses represent the 95% confidence intervals around the centroids of the respective species or body parts.

CHC profiles of the 16 species were highly species-specific (Figures 1A and 4A; PERMANOVA: pseudo-F_15_ = 98.5, *P = *0.0001). There were strong differences among the body parts (including PPG and whole-body profile; PERMANOVA: pseudo-F_4_ = 57.0, *P = *0.0001). Except for legs and thorax (*t* = 1.2, *P = *0.31), all body parts differed from each other (all pairwise *t* > 2.4, *P* ≤ 0.011). The whole-body profile was most similar to that of the PPG, followed by the gaster, whereas the legs were most different from the PPG (Figure 4B).

**Figure 5.**
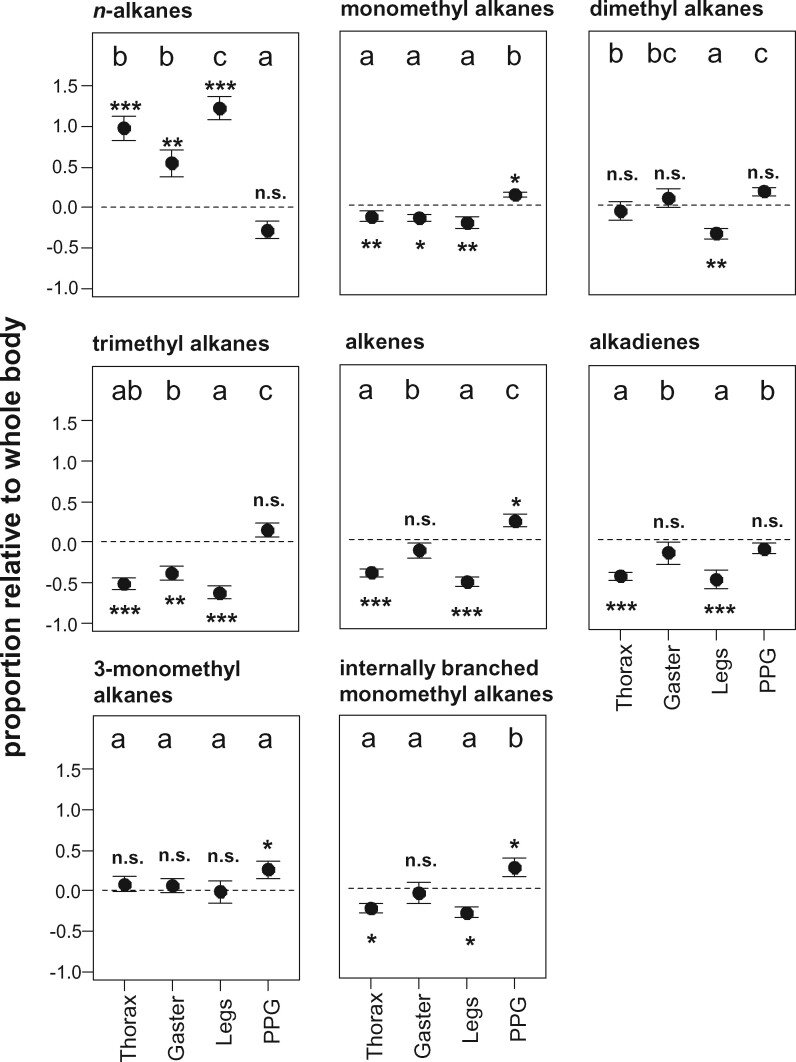
Body part-specific differences in CHC substance classes and homologous series among 16 different Central European ant species. Each plot shows means ± SE of the whole-body standardized proportion for 1 substance class or homologous series. Different letters indicate statistical differences among body parts based on the results of linear mixed effects models. Asterisks indicate statistical differences from the whole-body profile (deviation from 0).

To investigate the differences more closely, we tested if the proportions of certain substance classes or homologous series relative to the whole bodies differed among body parts, but also if the proportions on the body parts differed significantly from the whole-body CHC extracts. We found CHC differences among body parts in 9 out of 11 substance classes or homologous series (Table 1; Figure 5). Only tetramethyl alkanes (which only occurred in 1 species in the comparison, *Formica rufibarbis*) and 3-methyl alkanes did not differ in their relative proportions between legs, thoraces, gasters, and PPGs. In addition, we tested which of the body parts significantly differed from the whole-body profiles. Legs were nearly always different from the whole bodies: Although they contained nearly twice the proportion of *n*-alkanes compared with the whole body, they had lower proportions of mono-, di-, and trimethyl alkanes, alkenes, and alkadienes as well as 3, x- and internally-branched dimethyl alkanes (Figure 5; Supplementary Figure S1B; Supplementary Table S2). In PPGs, the effects were usually in the opposite direction: the PPG contained more alkenes as well as 3- and internally-branched monomethyl alkanes compared with the whole bodies (Figure 5; Supplementary Table S2). Although we found more *n*-alkanes on legs, thoraces, and gasters, they contained fewer monomethyl alkanes (Figure 5; Supplementary Table S2). Furthermore, legs and thoraces contained fewer alkenes and alkadienes than the whole body (Figure 5; Supplementary Table S2). The average chain length of *n*-alkanes did not differ among body parts (Table 1); only the *n*-alkanes of the gaster were shorter than those in the whole-body extracts (Supplementary Figure S2).

**Table 1.** Differences in proportions of CHC substance classes and homologous series of methyl alkanes between body parts in a comparison among 16 Central European ant species

Substance class	χ^2^	*df*	*P*-value	*n*
*n*-alkanes	89.30	3	**<0.0001**	16, 54
Monomethyl alkanes	29.26	3	**<0.0001**	16, 53
Dimethyl alkanes	33.20	3	**<0.0001**	16, 54
Trimethyl alkanes	76.51	3	**<0.0001**	8, 27
Tetramethyl alkanes	3.76	3	0.29	1, 4
Alkenes	74.13	3	**<0.0001**	14, 46
Alkadienes	14.38	3	**0.0024**	6, 21
3-methyl alkanes	4.99	3	0.17	14, 46
Internally-branched methyl alkanes	36.42	3	**<0.0001**	16, 54
3, x-dimethyl alkanes	24.96	3	**<0.0001**	10, 33
Internally-branched dimethyl alkanes	19.41	3	**0.00022**	12, 40
Chain length of *n-*alkanes	6.44	3	0.092	16, 54

The table gives the results of linear mixed effects models with the proportion per substance class or homologous series as dependent variable and the body parts as fixed effects. Significant *P*-values are printed in bold. Note that, because not all substance classes are present in all species, *n* gives the sample sizes for species (total 16) and individuals (total 54) used in the models.

**Figure 6.**
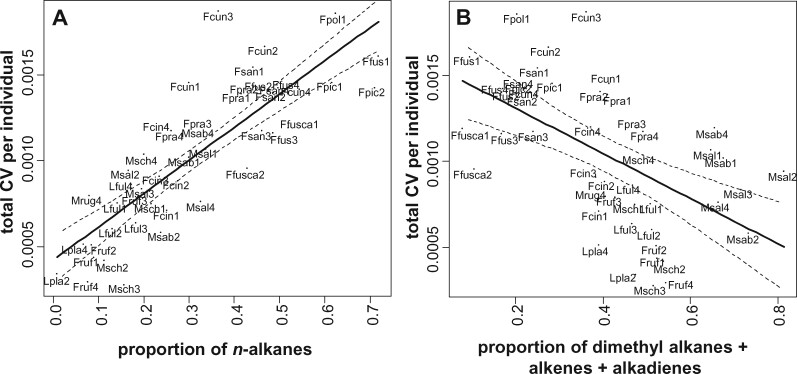
Total intraindividual CV in dependence of the proportion of *n*-alkanes (**A**) and most fluid CHC substance classes (**B**). Each dot represents 1 individual with the intraindividual CV on the *y*-axis and the proportion of *n*-alkanes (A) or the sum of dimethyl alkanes, alkenes, and alkadienes (B) on the *x*-axis. Regression lines indicate the positive or negative relationship between the intraindividual CV and the proportions. For the species abbreviations, see Figure 1A.

The total CV per individual (across CHC classes) increased with the proportion of *n*-alkanes in the CHC profile of the species (χ^2^_1_ = 59.92, *P < *0.0001; Figure 6A). With higher proportions of monomethyl alkanes but slightly not dimethyl alkanes the CV per individual decreased slightly (monomethyl alkanes: χ^2^_1_ = 4.27, *P = *0.03; dimethyl alkanes: χ^2^_1_ = 2.9, *P = *0.088; data not shown). However, the proportions of alkenes and alkadienes alone did not influence the CV per individual (alkenes: χ^2^_1_ = 1.48, *P = *0.22; alkadienes: χ^2^_1_ = 0.58, *P = *0.45; data not shown). In contrast, the sum of the most fluid substance classes (dimethyl alkanes, alkenes, and alkadienes) had a strong negative effect on the total CV per individual (χ^2^_1_ = 11.90, *P = *0.00056; Figure 6B).

## Discussion

Our results revealed consistent CHC variation among body parts across multiple species. This has not been reported before, because, in contrast to CHC variation among individuals or among species (Martin and Drijfhout 2009a; Blomquist and Bagnères 2010; Martin et al. 2013; Sprenger and Menzel 2020), only few studies dealt with variation among body parts (Gibbs and Crowe 1991; Young et al. 2000; Wang et al. 2016a, 2016b, 2019). However, such intraindividual variation means potential variation in information content, which can affect nestmate recognition and intracolonial communication.

Where does this variation stem from? One possible mechanism might involve the selective transport of CHCs to different parts of the cuticle as it has been shown before in moths, where long-chain aliphatic sex pheromones are selectively transported to certain glands (Schal et al. 1998a; Jurenka et al. 2003; Bagnères and Blomquist 2010). Unfortunately, to our knowledge, there are no studies on this question, for example, whether CHCs are transported from oenocytes to different parts of the cuticle in a nonrandom way. Thus, this idea cannot be tested up to now. Alternatively, differences may relate to physical differences between CHCs, that is, variation in melting point and viscosity. The observed effects are consistent with this hypothesis: ant species rich in early-melting CHCs like alkenes or dimethyl alkanes indeed had a lower intraindividual variability (Figure 6B), suggesting a higher CHC diffusion. In contrast, intraindividual variability increased with the proportion of *n*-alkanes (Figure 6A). This agrees with an earlier study, where *n*-alkanes applied to the elytra of potato beetles were relatively immobile, whereas alkenes diffused over the body and were later detectable in the tarsi of the beetles (Geiselhardt et al. 2010).

The strongest argument for a biophysical cause behind the observed body-part differences is that nearly all of them followed a consistent pattern: body parts varied from “rich in early-melting, rather liquid CHCs” to “rich in late-melting, rather solid CHCs” in the order PPG—whole body—gaster—thorax—legs (Figures 1B, 3, and 5). This fits the melting point variation among CHC classes, but also applies to differences within a CHC class, that is, the differentiation between terminally (3rd or 5th position) and internally-branched alkanes (Gibbs and Pomonis 1995; Gibbs 2002). Most notably, the PPG was richer in early-melting CHCs like di- and trimethyl alkanes, internally-branched monomethyl alkanes and alkenes, and poorer in CHCs with high melting points like *n-*alkanes, 5-monomethyl, and 3-monomethyl alkanes (Figures 3 and 5). Previous studies already reported higher *n-*alkane abundances on the cuticle compared with the PPG (Soroker and Hefetz 2000; Bagnères and Blomquist 2010). Compared with the PPG, the differences between the other body parts were less pronounced. Here, legs were richer in the relatively solid 3-monomethyl alkanes, *M. rubra*, (Figure 3B). Among the other species, legs had more *n-*alkanes (which are solid at ambient temperatures), and less of the relatively early-melting di- and trimethyl alkanes (Figure 5). Gaster and thorax were more similar (Figure 4), but the gaster carried more alkenes and alkadienes and less *n-*alkanes (Figure 5). The only exception to the described patterns was the alkenes in *M. rubra*, which, although early-melting, were highly correlated with the abundance of *n-*alkanes and hence less abundant in the PPG than in the other body parts (Figure 3A). This phenomenon still awaits explanation. It coincides with the tight correlation of *n-*alkanes and alkenes observed in *M. rubra* (Sprenger et al. 2018), and we found it only in this species, but not in the interspecific comparison.

The observed intraindividual variation may actually benefit the maintenance of different communication channels in the CHC profile. One problem of our current nestmate recognition model was how hydrocarbons could be exchanged and homogenized among colony members while maintaining differences between workers and queens, or between foragers and nurses (Leonhardt *et al.*, 2016). Biophysical differences between CHCs can explain this phenomenon: Nurses and foragers mainly differ in the proportion of *n-*alkanes (Wagner et al. 1998, 2001; Pamminger et al. 2014; Sprenger and Menzel 2020). This difference is maintained during CHC exchange if *n-*alkanes are taken up into the PPG to a smaller degree (or not at all). The same is true for other differences, for example, when internally-branched mono- or dimethyl alkanes are taken up to a higher degree than the less fluid terminally branched ones. Similarly, queen pheromones can persist on the queen despite CHC exchange if, during trophallaxis or grooming, they are exchanged to a lower degree than the other hydrocarbons. Queen pheromones usually have relatively long chains, and comprise *n-*alkanes or 3-monomethyl alkanes (e.g., 3-MeC31 and *n*-C31; Holman et al. 2010; Van Oystaeyen et al. 2014), which are the 2 substance groups with the highest melting temperatures. Hence, the fact that queen signals consist of relatively solid CHCs facilitates the maintenance of queen-worker differences despite CHC exchange. In contrast to caste or task-group differences, nestmate recognition cues seem to consist rather of early-melting CHCs. This is suggested by the fact that PPG extracts, which contain rather liquid and early-melting CHCs, were often shown to be sufficient for nestmate recognition (Martin et al. 2008; Guerrieri et al. 2009; Martin and Drijfhout 2009b; Sano et al. 2018). Thus, physical differences between CHCs allow the maintenance of independent information channels in a CHC profile with relatively little interference.

Why some body parts are richer in early- or late-melting CHCs is less clear. One possible cause is lower abrasion of solid hydrocarbons compared with liquid ones. In the legs, there is constant abrasion and hence a sink for liquid CHCs, which explains their high abundance of more solid CHCs like *n-*alkanes (Figure 5). More solid CHCs are beneficial for waterproofing, which is especially important on the legs due to their high surface-to-volume ratio. The high proportion of *n-*alkanes on legs can also explain the observation of Wang et al. (2019) that CHC profiles of legs are task-specific. Second, liquid CHCs should be exchanged to a higher degree than solid CHCs during allogrooming. Since legs are groomed less than other body parts like gaster or thorax, this would explain the higher proportion of liquid CHCs in gaster and thorax (Figure 5). Third, the fat body is located in the gaster. Thus, most newly produced CHCs should end up on the cuticle of the gaster rather than other body parts. From there they can diffuse to the remaining body parts (especially the liquid ones, but also the solid ones). These hypotheses can explain the observed CHC variation, but do not require selective transport of certain CHC classes. Thus, body-part differences may be caused by uneven, “regionalized” CHC secretion onto the body surface, followed by diffusion that differs among CHC classes.

To our knowledge, this is the first study that describes intraindividual CHC differences across many insect species. They form a consistent pattern of body parts richer in late-melting, rather solid CHCs and early-melting, rather liquid ones. Our results have important implications for communication in social insects: firstly, models of nestmate recognition that assume a homogenous odor across the insect body are not entirely correct. Rather, nestmate recognition cues are not homogenously distributed across the body surface, and might occur only in low abundances on the legs. Second, hydrocarbon exchange via trophallaxis and grooming is necessarily selective, because the more liquid CHCs are preferentially taken up and redistributed. However, this should actually be beneficial for communication, since it allows that differences between castes and task groups can be maintained despite CHC exchange. Nestmate recognition should not be hampered by uneven CHC diffusion if it relies mostly on early-melting, rather liquid CHCs, and should be possible for most body parts except possibly for the legs. Thus, physical differences between CHCs may represent a key mechanism that enables parallel information channels in insect communication. Future studies should address not only 1) the biosynthetic pathways that lead to body-part differentiation in CHC profiles, but also 2) the evolutionary trajectories of CHC signaling that led to information being encoded by early-melting or late-melting hydrocarbons.

## Supplementary Material

zoab012_Supplementary_Data

## References

[zoab012-B1] Bagnères AG, Blomquist GJ, 2010. Site of synthesis, mechanism of transport and selective deposition of hydrocarbons. In: Blomquist GJ, Bagnères AG, editors. Insect Hydrocarbons: Biology, Biochemistry, and Chemical Ecology. New York: Cambridge University Press. 75–99.

[zoab012-B2] Bagnères AG, Hanus R, 2015. Communication and social regulation in termites. In: Aquiloni L, Tricarico E, editors. Social Recognition in Invertebrates: The Knowns and the Unknowns. Basel: Springer International Publishing. 193–248.

[zoab012-B3] Blomquist GJ, Bagnères AG, 2010. Introduction: history and overview of insect hydrocarbons. In: Blomquist GJ, Bagnères AG, editors. Insect Hydrocarbons: Biology, Biochemistry, and Chemical Ecology. New York: Cambridge University Press. 3–18.

[zoab012-B4] Boulay R, Hefetz A, Soroker V, Lenoir A, 2000. *Camponotus fellah* colony integration: worker individuality necessitates frequent hydrocarbon exchanges. Anim Behav 59:1127–1133. 10877891 10.1006/anbe.2000.1408

[zoab012-B5] Carlson DA, Bernier UR, Sutton BD, 1998. Elution patterns from capillary GC for methyl-branched alkanes. J Chem Ecol 24:1845–1865.

[zoab012-B6] Fan Y, Schal C, Vargo EL, Bagnères AG, 2004. Characterization of termite lipophorin and its involvement in hydrocarbon transport. J Insect Physiol 50:609–620. 15234621 10.1016/j.jinsphys.2004.04.007

[zoab012-B7] Geiselhardt SF, Lamm S, Gack C, Peschke K, 2010. Interaction of liquid epicuticular hydrocarbons and tarsal adhesive secretion in *Leptinotarsa decemlineata* Say (Coleoptera: chrysomelidae). J Comp Physiol A 196:369–378. 10.1007/s00359-010-0522-8 20361192

[zoab012-B8] Gibbs AG, 1995. Physical properties of insect cuticular hydrocarbons: model mixtures and lipid interactions. Comp Biochem Phys B 112:667–672.

[zoab012-B9] Gibbs AG, 1998. The role of lipid physical properties in lipid barriers. Am Zool 38:268–279.

[zoab012-B10] Gibbs AG, 2002. Lipid melting and cuticular permeability: new insights into an old problem. J Insect Physiol 48:391–400. 12770088 10.1016/s0022-1910(02)00059-8

[zoab012-B11] Gibbs AG, Crowe JH, 1991. Intra-individual variation in cuticular lipids studied using Fourier transform infrared spectroscopy. J Insect Physiol 37:743–748.

[zoab012-B12] Gibbs AG, Pomonis JG, 1995. Physical properties of insect cuticular hydrocarbons: the effects of chain length, methyl-branching and unsaturation. Comp Biochem Phys B 112:243–249.

[zoab012-B13] Gibbs AG, Rajpurohit S, 2010. Cuticular lipids and water balance. In: Blomquist GJ, Bagnères AG, editors. Insect Hydrocarbons: Biology, Biochemistry, and Chemical Ecology. New York: Cambridge University Press. 100–120.

[zoab012-B14] Greene MJ, Gordon DM, 2003. Cuticular hydrocarbons inform task decisions. Nature 423:32. 12721617 10.1038/423032a

[zoab012-B15] Guerrieri FJ, Nehring V, Jørgensen CG, Nielsen J, Galizia CG et al, 2009. Ants recognize foes and not friends. Proc R Soc B 276:2461–2468. 10.1098/rspb.2008.1860 PMC2690455 19364750

[zoab012-B16] Helmkampf M, Cash E, Gadau J, 2015. Evolution of the insect desaturase gene family with an emphasis on social Hymenoptera. Mol Biol Evol 32:456–471. 25425561 10.1093/molbev/msu315 PMC4298175

[zoab012-B17] Holman L, Jørgensen CG, Nielsen J, d’Ettorre P, 2010. Identification of an ant queen pheromone regulating worker sterility. Proc R Soc B 277:3793–3800. 10.1098/rspb.2010.0984 PMC2992706 20591861

[zoab012-B18] Jurenka RA, Subchev M, Abad JL, Choi MJ, Fabrias G, 2003. Sex pheromone biosynthetic pathway for disparlure in the gypsy moth *Lymantria dispar*. Proc Nat Acad Sci USA 100:809–814. 12533665 10.1073/pnas.0236060100 PMC298683

[zoab012-B19] Lahav S, Soroker V, Hefetz A, Vander Meer RK, 1999. Direct behavioral evidence for hydrocarbons as ant recognition discriminators. Naturwissenschaften 86:246–249.

[zoab012-B20] LeBoeuf AC, Waridel P, Brent CS, GonçAlves AN, Menin L et al, 2016. Oral transfer of chemical cues, growth proteins and hormones in social insects. eLife 5:e20375. 27894417 10.7554/eLife.20375 PMC5153251

[zoab012-B21] Leonhardt SD, Menzel F, Nehring V, Schmitt T, 2016. Ecology and evolution of communication in social insects. Cell 164:1277–1287. 26967293 10.1016/j.cell.2016.01.035

[zoab012-B22] Liebig J, 2010. Hydrocarbon profiles indicate fertility and dominance status in ant, bee and wasp colonies. In: Blomquist GJ, Bagnères AG, editors. Insect Hydrocarbons: Biology, Biochemistry, and Chemical Ecology. New York: Cambridge University Press. 254–281.

[zoab012-B23] Martin SJ, Drijfhout FP, 2009a. A review of ant cuticular hydrocarbons. J Chem Ecol 35:1151–1161. 19866237 10.1007/s10886-009-9695-4

[zoab012-B24] Martin SJ, Drijfhout FP, 2009b. Nestmate and task cues are influenced and encoded differently within ant cuticular hydrocarbon profiles. J Chem Ecol 35:368–374. 19263166 10.1007/s10886-009-9612-x

[zoab012-B25] Martin SJ, Vitikainen E, Helanterä H, Drijfhout FP, 2008. Chemical basis of nest-mate discrimination in the ant *Formica exsecta*. Proc R Soc B 275:1271–1278. 10.1098/rspb.2007.1708 PMC2602672 18319215

[zoab012-B26] Martin SJ, Vitikainen E, Shemilt S, Drijfhout FP, Sundström L, 2013. Sources of variation in cuticular hydrocarbons in the ant *Formica exsecta*. J Chem Ecol 39:1415–1423. 24272518 10.1007/s10886-013-0366-0 PMC3851696

[zoab012-B28] Menzel F, Morsbach S, Martens JH, Räder P, Hadjaje S et al, 2019. Communication vs. waterproofing: the physics of insect cuticular hydrocarbons. J Exp Biol 222:jeb210807. 31704903 10.1242/jeb.210807

[zoab012-B29] Murdoch D, Chow ED, 2020. *Ellipse: Functions for Drawing Ellipses and Ellipse-Like Confidence Regions*. Available from: https://cran.R-project.org/package=ellipse (accessed 15 December 2020).

[zoab012-B30] Oksanen J, Blanchet FG, Friendly M, Kindt R, Legendre P et al, 2019. *vegan: Community Ecology Package*. Available from: https://cran.R-project.org/package=vegan (accessed 15 December 2020).

[zoab012-B31] Pamminger T, Foitzik S, Kaufmann KC, Schützler N, Menzel F, 2014. Worker personality and its association with spatially structured division of labor. PLoS ONE 9:e79616. 24497911 10.1371/journal.pone.0079616 PMC3907378

[zoab012-B32] Pinheiro J, Bates D, DebRoy S, Sarkar D, R Core Team, 2016. *nlme: Linear and Nonlinear Mixed Effects Models*. Available from: https://cran.R-project.org/package=nlme (accessed 15 December 2020).

[zoab012-B33] R Core Team, 2020. R: A Language and Environment for Statistical Computing. Vienna: R Found Stat Comput. http://www.R-project.org/ (accessed 15 December 2020).

[zoab012-B34] Rourke BC, Gibbs AG, 1999. Effects of lipid phase transitions on cuticular permeability: model membrane an in situ studies. J Exp Biol 202:3255–3262. 10539973 10.1242/jeb.202.22.3255

[zoab012-B35] Sano K, Bannon N, Greene MJ, 2018. Pavement ant workers *Tetramorium caespitum* assess cues coded in cuticular hydrocarbons to recognize conspecific and heterospecific non-nestmate ants. J Insect Behav 31:186–199.

[zoab012-B36] Schal C, Sevala VL, Cardé RT, 1998a. Novel and higly specific transport of volatile sex pheromone by hemolymph lipophorin in moths. Naturwissenschaften 85:339–342.

[zoab012-B37] Schal C, Sevala VL, Young HP, Bachmann JAS, 1998b. Sites of synthesis and transport pathways of insect hydrocarbons: cuticle and ovary as target tissues. Am Zool 38:382–393.

[zoab012-B38] Seifert B, 2007. Die Ameisen Mittel- Und Nordeuropas. Görlitz: lutra Verlags- und Vertriebsgesellschaft.

[zoab012-B40] Soroker V, Hefetz A, 2000. Hydrocarbon site of synthesis and circulation in the desert ant *Cataglyphis niger*. J Insect Physiol 46:1097–1102. 10817835 10.1016/s0022-1910(99)00219-x

[zoab012-B42] Soroker V, Vienne C, Hefetz A, 1995. Hydrocarbon dynamics within and between nestmates in *Cataglyphis niger* (Hymenoptera, Formicidae). J Chem Ecol 21:365–378. 24234067 10.1007/BF02036724

[zoab012-B43] Soroker V, Vienne C, Hefetz A, Nowbahari E, 1994. The postpharyngeal gland as a “Gestalt” organ for nestmate recognition in the ant *Cataglyphis niger*. Naturwissenschaften 81:510–513.

[zoab012-B44] Sprenger PP, Burkert LH, Abou B, Federle W, Menzel F, 2018. Coping with climate: cuticular hydrocarbon acclimation of ants under constant and fluctuating conditions. J Exp Biol 221:jeb171488. 29615527 10.1242/jeb.171488

[zoab012-B45] Sprenger PP, Menzel F, 2020. Cuticular hydrocarbons in ants (Hymenoptera: Formicidae) and other insects: how and why they differ among individuals, colonies and species. Myrmecol News 30:1–26.

[zoab012-B46] Steiger S, Ower GD, Stökl J, Mitchell C, Hunt J et al, 2013. Sexual selection on cuticular hydrocarbons of male sagebrush crickets in the wild. Proc R Soc B 280:20132353. 10.1098/rspb.2013.2353 PMC3826231 24197415

[zoab012-B47] Steiger S, Stökl J, 2014. The role of sexual selection in the evolution of chemical signals in insects. Insects 5:423–438. 26462692 10.3390/insects5020423 PMC4592599

[zoab012-B49] Van Oystaeyen A, Oliveira RC, Holman L, van Zweden JS, Romero C et al, 2014. Conserved class of queen pheromones stops social insect workers from reproducing. Science 343:287–290. 24436417 10.1126/science.1244899

[zoab012-B50] van Zweden JS, d’Ettorre P, 2010. Nestmate recognition in social insects and the role of hydrocarbons. In: Blomquist GJ, Bagnères AG, editors. Insect Hydrocarbons: Biology, Biochemistry, and Chemical Ecology. New York: Cambridge University Press. 222–243.

[zoab012-B51] Wagner D, Brown MJF, Broun P, Cuevas W, Moses LE et al, 1998. Task-related differences in the cuticular hydrocarbon composition of harvester ants *Pogonomyrmex barbatus*. J Chem Ecol 24:2021–2037.

[zoab012-B52] Wagner D, Tissot M, Gordon DM, 2001. Task-related environment alters the cuticular hydrocarbon composition of harvester ants. J Chem Ecol 27:1805–1819. 11545372 10.1023/a:1010408725464

[zoab012-B53] Wang Q, Goodger JQD, Woodrow IE, Chang L, Elgar MA, 2019. Task-specific recognition signals are located on the legs in a social insect. Front Ecol Evol 7:227.

[zoab012-B54] Wang Q, Goodger JQD, Woodrow IE, Elgar MA, 2016a. Location-specific cuticular hydrocarbon signals in a social insect. Proc R Soc B 283:20160310. 10.1098/rspb.2016.0310 PMC4822474 27030418

[zoab012-B55] Wang Y, Carballo RG, Moussian B, 2017. Double cuticle barrier in two global pests, the whitefly *Trialeurodes vaporariorum* and the bedbug *Cimex lectularius*. J Exp Biol 220:1396–1399. 28167802 10.1242/jeb.156679

[zoab012-B56] Wang Y, Yu Z, Zhang J, Moussian B, 2016b. Regionalization of surface lipids in insects. Proc R Soc B 283:20152994. 10.1098/rspb.2015.2994 PMC4874700 27170708

[zoab012-B57] Young HP, Larabee JK, Gibbs AG, Schal C, 2000. Relationship between tissue-specific hydrocarbon profiles and lipid melting temperatures in the cockroach *Blattella germanica*. J Chem Ecol 26:1245–1263.

