# Supplementary material for: Cuticular hydrocarbon profiles differ between ant body parts: implications for communication and our understanding of CHC diffusion
Source: Curr Zool. 2021 Feb 11;67(5):531–40. doi: 10.1093/cz/zoab012 (PMC8489164; doi:10.1093/cz/zoab012)
Supplement: zoab012_Supplementary_Data [file zoab012_supplementary_data.pdf]

Supplementary Material of "Interspecific comparison reveals conserved cuticular hydrocarbon differences between body parts of Central European ants"

Sprenger PP, Gerbes LJ, Sahm J, Menzel F

Table S1: Sample sizes and origins of examined species. The table shows sample sizes and collection sites (A – botanical gardens of the University of Bayreuth, Germany; B – Ober-Olmer forest near Mainz, Germany; C – Mainz, Germany; D – swampy meadow near Eupen, Belgium; E – forest near Darmstadt, Germany) per examined species. Unless noted otherwise, we used each individual for body-part extracts to obtain an extract each of PPG, gaster (G), legs (L) and thorax (T).

species	abbreviation	no. of individuals for whole-body extracts	no. of individuals for body-parts extracts)	collection site	collected by
Formica cinerea	*F. cin.*	3	4	A	M. Grevé
Formica cunicularia	*F. cun.*	3	4	A	M. Grevé
Formica fusca	*F. fusca*	2	2	A	M. Grevé
Formica fuscocinerea	*F. fus.*	3	4	A	M. Grevé
Formica picea	*F. pic.*	1	2	A	M. Grevé
Formica polyctena	*F. pol.*	1	1	B	F. Menzel
Formica pratensis	*F. pra.*	3	4	A	M. Grevé
Formica rufibarbis	*F. ruf.*	3	4	A	M. Grevé
Formica sanguinea	*F. san.*	3	4	A	M. Grevé
Lasius brunneus	*L. bru.*	1	1[*]	C	F. Menzel
Lasius fuliginosus	*L. ful.*	3	4	A	M. Grevé
Lasius platythorax	*L. pla.*	3	4[§]	D	F. Menzel
Myrmica rubra	*M. rub.*	0	22[$]	B	J. Sahm
Myrmica ruginodis	*M. rug.*	4	4[^]	E	F. Rosumek
Myrmica sabuleti	*M. sab.*	3	4[°]	A	M. Grevé
Myrmica salina	*M. sal.*	3	4	A	M. Grevé
Myrmica schencki	*M. sch.*	3	4	A	M. Grevé

[*] no samples for L; [§] 2 samples for L; [^] 3 samples for G, 2 samples for T and 1 sample for L; [°] 3 samples for T, G and L; [$] analysed independently of the interspecific comparison

Table S2: Relative differences between body parts and whole-body CHC profiles per substance class. The table shows the results of linear mixed effects models with the standardized proportions per substance class as dependent variable testing the difference from zero (difference towards whole-body). Negative t-values indicate lower proportions in the body part than in the whole-body. Significant p-values are printed in bold.

***n*-alkanes**	**t**	**p**		**alkenes**	**t**	**p**
legs	7.24	**< 0.0001**		legs	-6.31	**< 0.0001**
gaster	3.28	**0.0013**		gaster	-1.68	0.095
PPG	-1.72	0.087		PPG	3.08	**0.0025**
thorax	5.85	**< 0.0001**		thorax	-5.02	**< 0.0001**
**monomethyl alkanes**	**t**	**p**		**alkadienes**	**t**	**p**
legs	-2.99	**0.0033**		legs	-4.18	**0.0001**
gaster	-2.51	**0.013**		gaster	-1.44	0.16
PPG	1.49	0.14		PPG	-0.99	0.33
thorax	-2.22	**0.028**		thorax	-4.17	**0.0001**
**dimethyl alkanes**	**t**	**p**		**3-methyl alkanes**	**t**	**p**
legs	-2.94	**0.0038**		legs	-0.15	0.88
gaster	0.36	0.72		gaster	0.45	0.66
PPG	1.19	0.24		PPG	2.41	**0.017**
thorax	-0.94	0.35		thorax	0.82	0.41
**trimethyl alkanes**	**t**	**p**		**internally branched methyl alkanes**	**t**	**p**
legs	-5.79	**< 0.0001**		legs	-1.91	0.058
gaster	-3.87	**0.0002**		gaster	-0.64	0.53
PPG	0.71	0.48		PPG	2.42	**0.017**
thorax	-4.97	**< 0.0001**		thorax	-1.53	0.13
**tetramethyl alkanes**	**t**	**p**		**3,x-dimethyl alkanes**	**t**	**p**
legs	-0.20	0.84		legs	-3.52	**0.0007**
gaster	0.97	0.36		gaster	-0.55	0.59
PPG	0.70	0.50		PPG	1.05	0.30
thorax	0.40	0.70		thorax	-1.38	0.17
**chain length of *n*-alkanes**	**t**	**p**		**internally branched dimethyl alkanes**	**t**	**p**
legs	-0.53	0.60		legs	-2.87	**0.0050**
gaster	-2.92	**0.0039**		gaster	0.19	0.85
PPG	-0.95	0.34		PPG	1.30	0.20
thorax	-1.24	0.22		thorax	-1.21	0.23

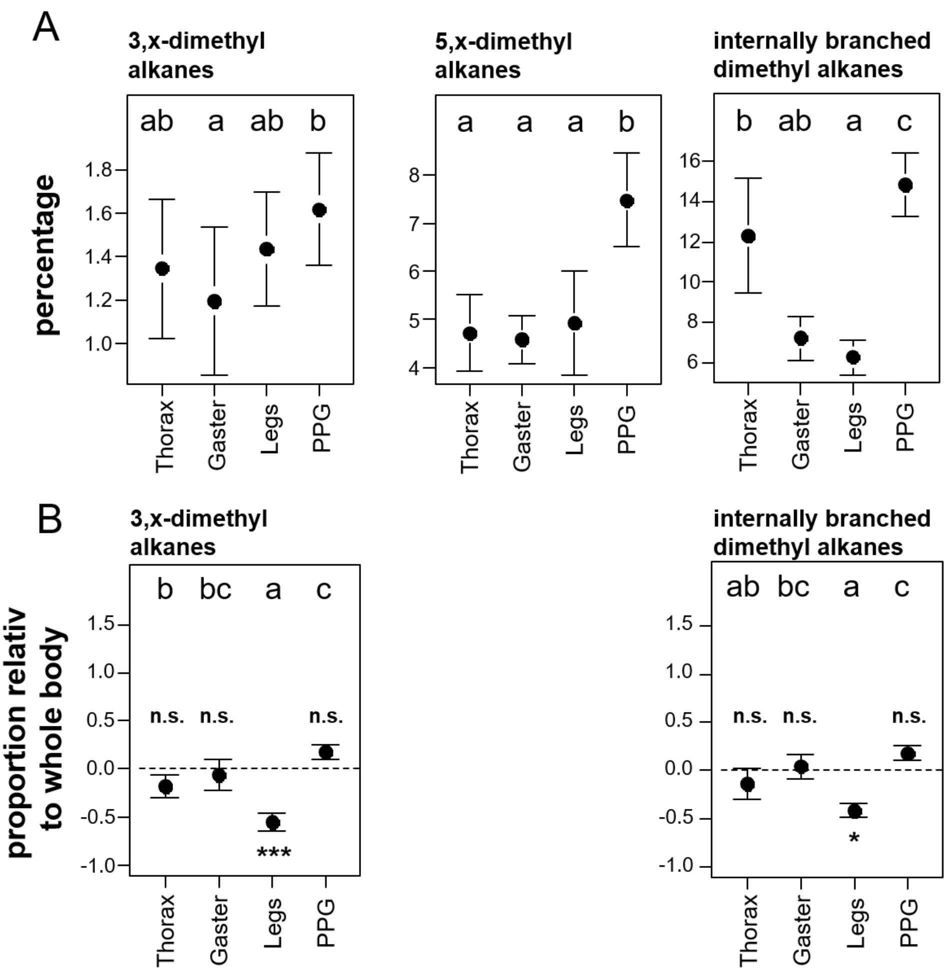

Figure S1: Differences between body parts in the homologous series of dimethyl alkanes in *Myrmica rubra* (A) and an interspecific comparison of 16 Central European ant species (B). In (A) each plot shows means ± SE of the proportion of one homologous series per body part in *M. rubra*. In (B) each plot shows means ± SE of the whole-body standardized proportion for one homologous series. Different letters indicate statistic differences among body parts based on the results of linear mixed effects models. Asterisks indicate statistical differences from the whole-body profile (deviation from zero).

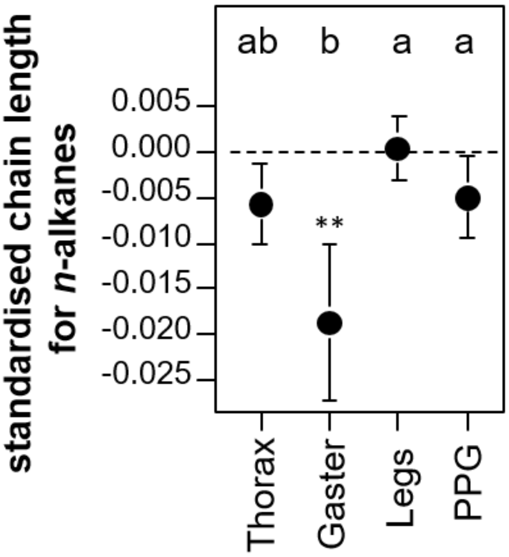

Figure S2: Differences in the standardised chain length for *n*-alkanes. The plot shows means ± SE. Different letters indicate statistic differences among body parts based on the results of linear mixed effects models. Asterisks indicate statistical differences from the whole-body profile (deviation from zero).
